# Functional Investigation of NCI-H460-Inducible Myofibroblasts on the Chemoresistance to VP-16 with a Microfluidic 3D Co-Culture Device

**DOI:** 10.1371/journal.pone.0061754

**Published:** 2013-04-16

**Authors:** Yuanyuan Hao, Lichuan Zhang, Jiarui He, Zhe Guo, Li Ying, Zhiyun Xu, Jianing Zhang, Jianxin Lu, Qi Wang

**Affiliations:** 1 Dalian Medical University, Dalian, P.R. China; 2 Department of Respiratory Medicine, Affiliated Zhongshan Hospital of Dalian University, Dalian, P.R. China; 3 Department of Respiratory Medicine, The Second Hospital Affiliated to Dalian Medical University, Dalian, P.R. China; 4 Department of Biochemistry, Institute of Glycobiology, Dalian Medical University, Dalian, P.R. China; 5 Key Laboratory of Laboratory Medicine, Ministry of Education, Zhejiang Provincial Key Laboratory of Medical Genetics, Wenzhou Medical College, Wenzhou, Zhejiang, P.R. China; University of Catania, Italy

## Abstract

Fibroblasts, the major cell type in tumor stroma, are essential for tumor growth and survival, and represent an important therapeutic target for cancers. Here we presented a microfluidic co-culture device in which the three-dimensional (3D) matrix was employed to reconstruct an in vivo-like fibroblast-tumor cell microenvironment for investigation of the role of myofibroblasts induced by lung cancer cells in the chemoresistance to VP-16. Composed of a double-layer chip and an injection pump, the device houses fibroblasts and lung cancer cells co-cultured in 3D matrix and 2D mode to induce fibroblasts to become myofibroblasts with the supplement of the medium continuously. With this device, we verified that the cytokines secreted by lung cancer cells could effectively transform the fibroblasts into myofibroblasts. Moreover, compared to fibroblasts, the myofibroblasts showed higher resistance to anticancer drug VP-16. We also demonstrated that this kind of acquired resistance in myofibroblasts was associated with the expression of Glucose-regulated protein 78 (GP78). We concluded that this device allows for the assay to characterize various cellular events in a single device sequentially, facilitating a better understanding of the interactions among heterotypic cells in a sophisticated microenvironment.

## Introduction

Lung cancer is the leading cause of the cancer mortality worldwide. Up to now, Chemotherapy is still a primary clinical strategy to treat lung cancers. However, chemoresiatance impedes therapeutic outcome and represents a major obstacle in cancer therapy [1]. Despite the fact that many intrinsic mechanisms associated with chemoresistance have been studied and identified, factors promoting drug resistance remain poorly defined. Cancer cells closely interact with their surrounding stromal compartment composed of fibroblasts, inflammatory cells, extracellular matrix and vessels. Fibroblasts make up the largest number of stroma cells and could be influenced by the tumor cells through secreted soluble factors [2], [3]. Once activated, they were then termed as cancer-associated fibroblasts or myofibroblasts, which will differ from normal fibroblasts greatly [2], [4]. Myofibroblasts are key mediators of tumor growth including proliferation, invasion and metastasis, and can contribute to chemoresistance [5], [6], [7]. However, the response and role of the myofibroblasts in chemoresistance are still unclear. Thus, there is an eager demand to establish new therapies based on eliminating these cells to improve the efficacy of cancer chemotherapy.

In a typical tumor microenvironment, cell-to-cell communication can be classified at least into two different modes: direct and indirect contact. In the direct mode, cells communicate through the direct cell-to-cell contact. In the indirect mode, cells communicate through the diffusible signals released by the cells in near vicinity. Till now, many studies have been focused on the direct mode, but rare addressed the indirect mode. In this article, we investigated the influence of lung cancer cells on the formation of myofibroblasts in the indirect contact mode with the novel microfluidic co-culture device. Also, in order to observe the activation of fibroblasts induced by cancer cells through diffusible signals, the cell-to-cell direct contact should be strictly avoided; thereby the sole effect from the indirect contact mode on cellular phenotype becomes measurable and understandable. One way to achieve this goal is to utilize a co-culture setup for in vitro assays. Although a few methods such as Transwell assay [8] or bathing cells in conditioned medium [9] were proposed to study the interactions between heterotypic cells, utilization of these methods are still limited because of the static instrumental set-up, poor flexibility, and tedious manual operations. More specifically, with these devices, it is difficult to control the microenvironment, which is important for cell communication.

Recently, microfluidics has attracted significant attention in cell-based biological and medical research, due to its miniaturized size, low consumption of reagents, and capabilities to integrate manifold experimental operation units onto a single device [10], [11]. A number of microfluidic devices have been implemented to study cell-cell interaction [12], [13], [14], [15]. Such devices are particularly useful for analyzing complicated cellular responses with well-controlled spatial and transient parameters. Moreover, the cell-to-volume ratios in the microfluidic devices are usually higher than those in conventional cell culture dishes, making it easy to observe and quantify cellular response. [14], [16].

We developed a double-layer microfluidic co-culture device to recreate an in vivo-like 3D tumor microenvironment for fibroblasts. In this device, lung cancer cells were cultured in the upstream in 2D mode and were allowed to secrete soluble factors to feed the fibroblasts co-cultured downstream in 3D mode. Fibroblasts were thus induced to transform into myofibroblasts via indirect contact. With this new platform, we are able to analyze the function of the myofibroblasts in the chemoresistance to anticancer drug VP-16 and its possible mechanism associated with a representative family member of Glucose-regulated protein, GRP78.

## Materials and Methods

### Design and Fabrication of the Microfluidic Co-culture Device

**Figure 1:**
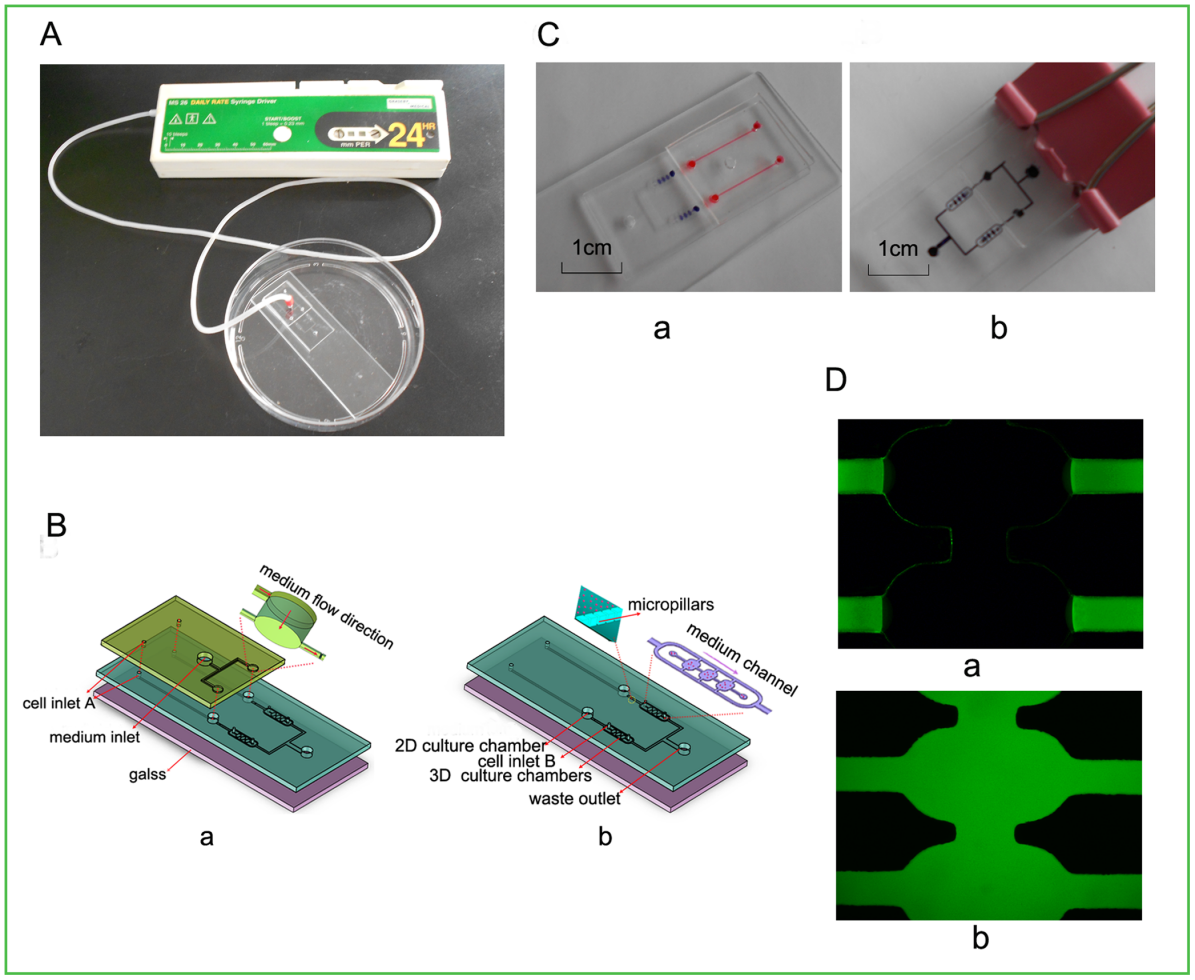
Microfluidic co-culture device design. (A) Image of the microfluidic device mainly composed of a double-layer chip and an injection pump. (B) Schema chart of the double-layer chip: (a)-(b) the layout of each layer. (C) Photograph of medium flow direction in the chip. (a) Injection of red and blue indicators from inlet A and B representing two types of cells, respectively, to demonstrate indirect contact co-culture. (b) Injection of black indicator from medium inlet to demonstrate medium injection. A simple external small clip was served as micro-valves to facilitate the medium flowing downstream. (D) The diffusion of FITC-Dextran in 3D matrix. (a) After 5 min. (b) After 60 min. Magnification: ×100.

The microfluidic device was composed of a double-layer chip and an injection pump (Fig. 1A). The upper layer of the double-layer chip was fabricated in PDMS (Sylgard 184, Dow Corning, Midland, MI, USA) by standard soft lithography methods [17] and was mainly used to supply the cells with flow fresh medium through a syringe pump mimicking the microenvironment in vivo. As shown in Fig. 1B (a), three inlets of 2 mm diameter were drilled on this layer, with one inlet for medium loading and the other two inlets for cell loading. The lower layer (Fig. 1B (b)) was fabricated in PDMS by dry etching methods [18], and was irreversibly bonded to a glass slide assisted by oxygen plasma surface treatment. This layer consisted of two separated co-culture units for the culture of two groups of cells in parallel simultaneously. Each co-culture unit mainly consisted of a long microchannel (15 mm×300 µm×50 µm), an upstream 2D cell culture chamber (1 mm diameter), a series of micro-pillars and a row of three downstream 3D cell culture chambers (800 µm×400 µm×50 µm each). On micro-channel, a small clip was employed as a micro-valve after cell loading to facilitate the medium flowing downstream. Between the upstream of the 2D culture chamber and the 3D culture chambers, a series of micro-pillars with the micro-gaps (8 µm) less than cells were placed to block the cells cross from the upstream and as a result, only the soluble secreted from the cells could move through to the downstream of 3D cell culture chambers. Finally, the two layers were aligned and combined together.

### Cell Culture

In our study, the human non-small cell lung cancer cell line NCI-H460 and the human fetal fibroblast cell line HFL1 were used to test the paracrine loop of lung cancers and its function on the fibroblasts. The two types of cells were obtained from the Cell Bank of Type Culture Collection of Chinese Academy of Sciences (Shanghai, China) and were maintained in RPMI-1640 medium (Sigma), supplemented with 10% fetal bovine serum (HyClone) and 1% antibiotic (santibiotic pen-strep-ampho) at 37°C with 5% CO_2_ and 95% relative humidity.

### Co-culture of NCI-H460 in 2D and HFL1 in 3D on the Microfluidic Device to Induce the Fibroblasts to be Myofibroblasts

In order to test whether the lung cancer cells can induce the fibroblasts to be myofibroblasts, the fibroblasts were divided into experimental group and control group. In the experimental group, the lung cancer cells and the fibroblasts were co-cultured in 2D and 3D mode, respectively; in the control group, only the fibroblasts were cultured in 3D mode with the medium supply from the upstream 2D cell culture chamber. Briefly, the suspension of the NCI-H460 cells in medium (10^6^ cells/mL) was infused into the 2D cell culture chamber through inlet A. cells were allowed to attach and secrete cytokine for 12 h, with the micro-pillar array to prevent the cells to flow across to the downstream 3D cell culture chambers. Then, the HFL1 cells were trypsinized, and embedded at 10^6^ cells/ml in the 0.24% type I collagen gel solution (pH 7; Cell Matrix Type Ia collagen; Koken) at 4°C. Cell-gel mixture was patterned into the 3D cell culture chambers through inlet B to be cultured with the supply of the cytokine secreted by the lung cells from the upstream of 2D cell culture chamber. During the whole process, an injection pump was used to connect with the medium inlet in the upper layer of the chip by an air-permeable silicone tube and supply the cells with fresh medium continuously at a flow rate of 1 mm/24 h [19]. The device was then moved to the cell culture incubator and stored for 4 days. Then, the viabilities of the two types of cells were detected by a Trypan blue exclusive assay. Mainly, the cells were exposed to 0.2% Trypan blue solution and immediately observed under an inverse light microscope. To verify the soluble factors secreted by lung cancer cells could diffuse into the 3D matrix via medium, the diffusion of FITC-Dextran (Sigma, MW 20,000 Da, with similar size to those of cytokines secreted by cancer cells) in the collagen gel was observed. FITC-Dextran was injected via medium inlets and the images of FITC-Dextran diffusion in the collagen gel were collected by the Confocal System (Nikon EZ-C1, Tokyo, Japan).

### α-SMA and GRP78 Assay on HFL1 Cells by Immunofluorescence Imaging

Expression of α-smooth muscle actin (α-SMA), a well accepted marker for myofibrobalsts, was detected and used to examine the effect of induction by cancer cells [2], [4]. Briefly, after co-culture for 4 days, HFL1 cells were fixed in 4% paraformaldehyde for 20 min, permeabilized in PBS containing 0.1% Triton X-100 and 5% bovine serum albumin for 30 min. Then, the cells were incubated with primary antibody (α-SMA: Abcam, 1∶400) over night. The next day, they were rinsed with PBS for twice, incubated with FITC green-conjugated secondary antibody (Santa Cruz Biotechnology, 1∶200) for 1 h. Images were subsequently captured using a Confocal microscope and analyzed using the Image-Pro Plus Imaging software (version 6.0; Media Cybernetics, USA).

In order to investigate whether stress response was involved in the myofibroblast transformation induced by lung cancer cells, the expression of GRP78, a typical stress protein, was detected in HFL1 cells. A similar procedure as above was performed using mouse polyclonal GRP78 antibody (Santa Cruz Biotechnology, 1∶400), and an anti-mouse (Santa Cruz Biotechnology, 1∶200) secondary antibody.

### Apoptosis Assay on HFL1 Cells after being Treated by VP-16

To elucidate whether myofibroblasts show chemoresistance to anticancer drug, the apoptosis of HFL1 cells was analyzed after treatment with different concentrations of VP-16 varying from 0 to 60 µM. The drug medium with VP-16 was introduced into the culture chambers via medium inlet. After 12 h incubation, the cells were washed with PBS. Hoechst of 5 µg/mL was injected into the microchambers and incubated for 30 min, rinsed by PBS for twice, and then cells were stained with 10 µg/mL propidium iodide (PI). Stained nuclei were observed and photographed with alive cells being stained blue whereas apoptotic cells being stained red [19], [20].

To examine the function of GRP78 in the chemotherapy resistance to VP-16, (-)-Epigallocatechin gallate (EGCG), a known GRP78 inhibitor [21], was used in the experimental group HFL1 cells. Briefly, the myofibroblasts were divided into EGCG-pretreated group and non-EGCG-pretreated group. The former was exposed to the medium containing EGCG (20 µM) and the latter was subjected to free medium (absent of inhibitor). 4 h later, the two groups of cells were exposed to VP-16 (0–60 µM) again and the apoptosis of cells was analyzed. Hoechst of 5 µg/mL and PI of 10 µg/mL were used to label alive cells and apoptotic cells.

## Results

### Fabrication of the Microfluidic Device and Cell Culture

A double-layer chip was designed and made successfully for cell co-culture. Indicators with different colors were used to validate the co-culture of two types of cells on this device with indirect contact. As shown in Fig. 1C, when the red and blue indicators were injected into the lower layer of the microchip through inlets A and B, respectively, the media could spread out to upstream 2D cell chamber and downstream 3D cell chambers uniformly; when the black indicator was injected into the upper layer of the microchip via medium inlet, the medium could flow into all the cell chambers of lower layer smoothly. For the assay of the cell viability on the device no matter in 2D or 3D, Trypan blue exclusion assay showed less than 2% of cells died. All these indicated that the microfluidic system was practicable for co-culture of two types of cells and provided a suitable environment for the cell growth.

### The Diffusion of FITC-Dextran into the Collagen Gel

In this work we aimed to establish a device to keep the co-culture medium flowed through the upstream 2D culture chamber and diffused into the downstream 3D chambers to deliver nutrients. In order to verify the soluble factors secreted by lung cancer cells could diffuse into the 3D matrix via medium, the diffusion of FITC-Dextran was assayed in the collagen gel. Fig. 1D depicted the diffusion of FITC-Dextran into the collagen gel after being injected for 5 and 60 min, respectively.

### Activation of Fibroblasts in Co-culture with Lung Cancer Cells

**Figure 2:**
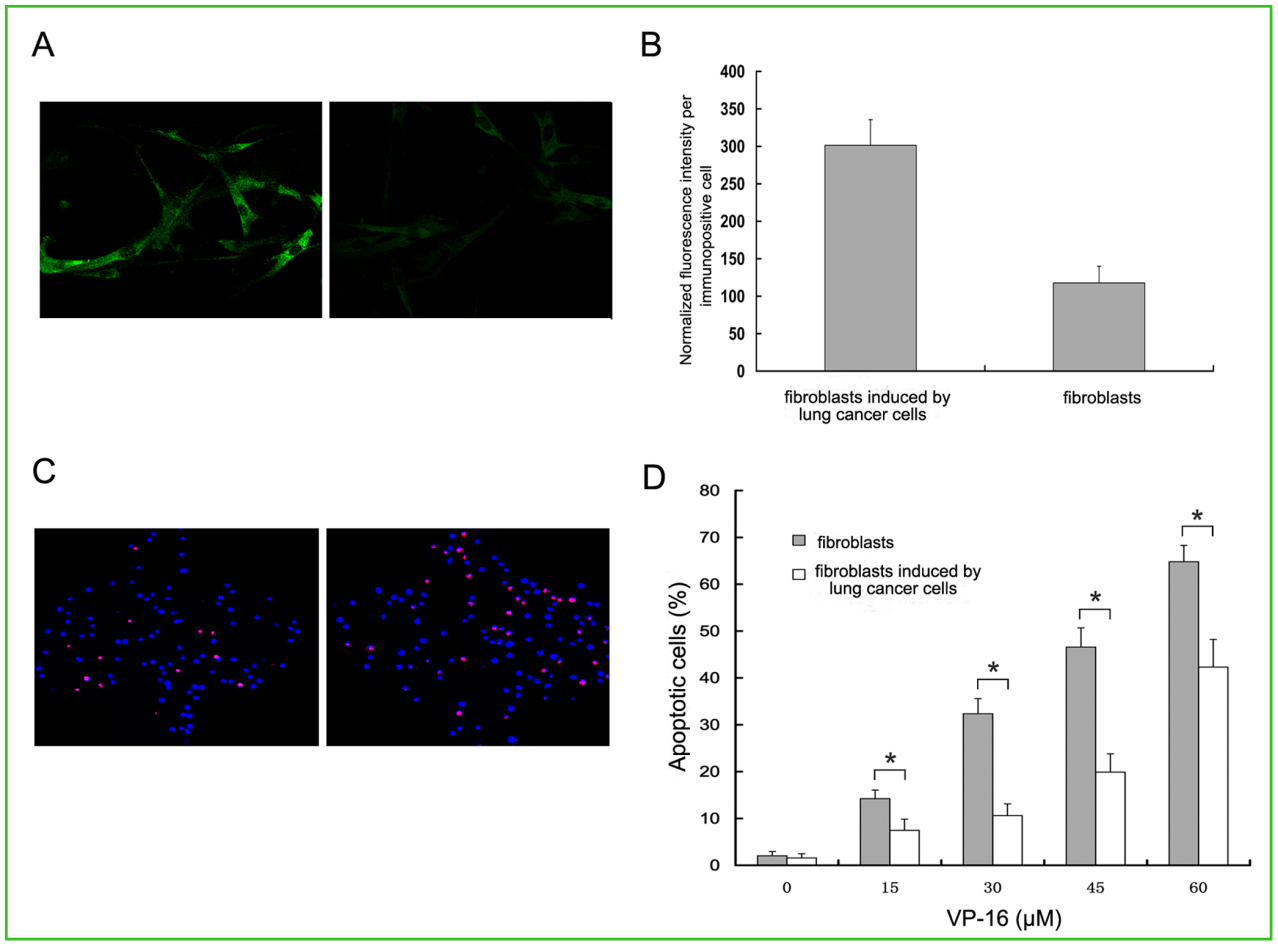
Analysis of α-SMA expression and measurement of apoptosis in fibroblasts induced and non-induced by lung cancer cells. (A) α-SMA protein assay by immunofluorescence imaging on fibroblasts induced and non-induced by lung cancer cells. (a) Induced. (b) Non-induced. Magnification: ×600. (B) The average expression of α-SMA in per cell was reflected by normalized fluorescent intensity. Data were shown as mean±SD of triplicate determinations. (C) Fluorescent analysis of apoptosis in fibroblasts induced and non-induced by lung cancer cells with PI and Hoechst after treatment with VP-16 (30 µM). Magnification: ×100. (D) The statistic analysis of percentage of apoptotic cells induced and non-induced by the lung cancer cells after treatment with different concentrations of VP-16 (0–60 µM). *p<0.05 compared with the control group. All the experiments were repeated at least three times.

In order to detected whether the lung cancer cells can activate fibroblasts and turn them into myofibroblasts, we carried out an immunoflurescence assay on the fibroblasts with or without the induction by the lung cancer cells (experimental and control group cells). As shown in Fig. 2A and Fig. 2B, the results showed that the α-SMA was expressed abundantly in the experimental group. However, only mild α-SMA expression was detected in the non-induced cells (p<0.05). This finding suggested that the cytokines secreted by cancer cells were able to activate the fibroblasts into myofibrobaslts.

### GRP78 Expression in Myofibroblasts

**Figure 3:**
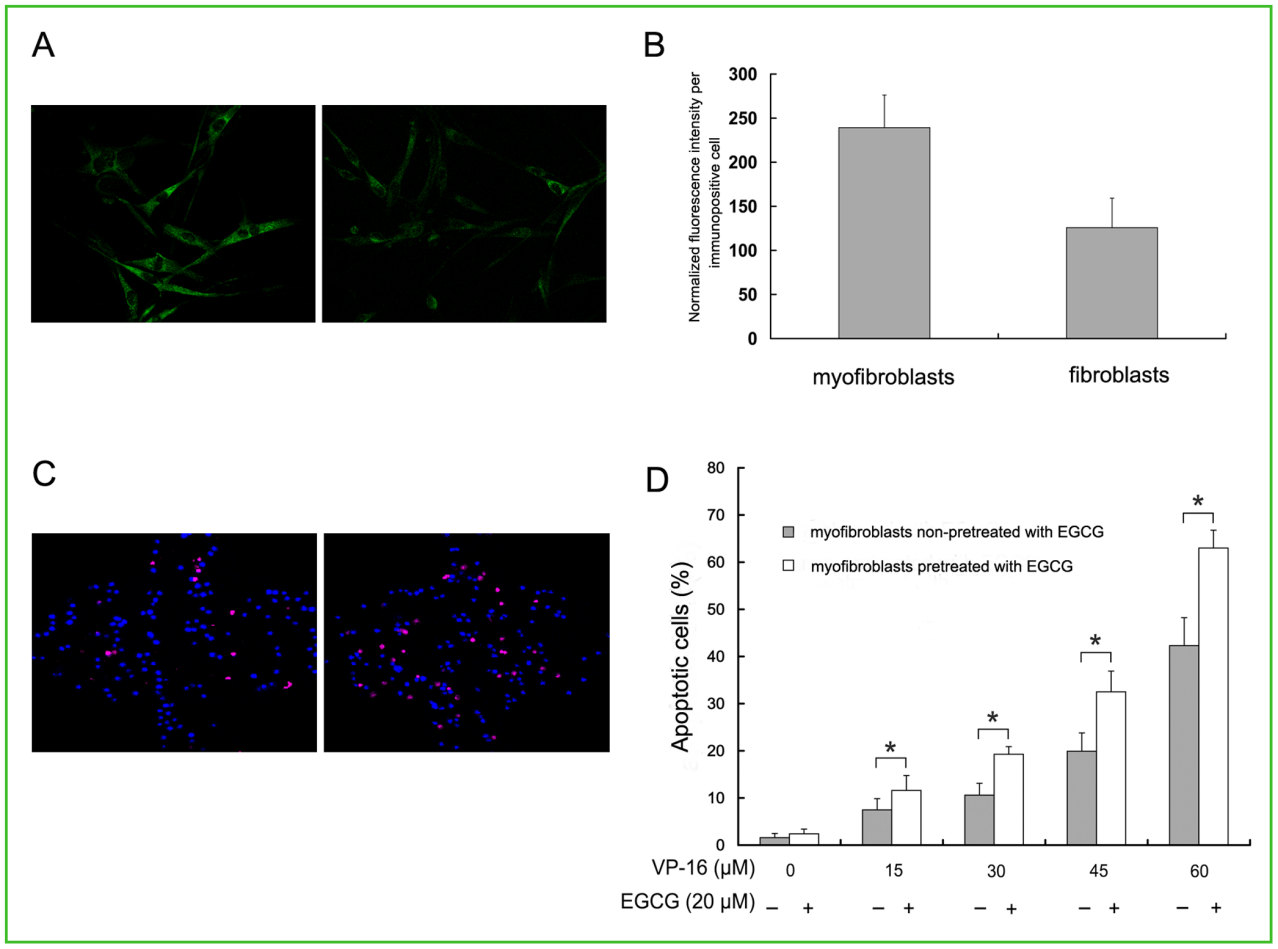
Analysis of GRP78 expression and effect of EGCG on VP-16 induced apoptosis in myofibroblasts. (A) GRP78 protein assay by immunofluorescence imaging on (a) myofibroblasts and (b) fibroblasts. Magnification: ×600. (B) The average expression of GRP78 in per cell was reflected by normalized fluorescent intensity. Data were shown as mean±SD of triplicate determinations. (C) Fluorescent analysis of percentage of apoptotic cells for myofibroblasts by EGCG pretreated and non-EGCG pretreated groups after treatment with VP-16 (30 µM). Magnification: ×100. (D) The statistic analysis of percentage of apoptotic cells for myofibroblasts in EGCG pretreated and non-EGCG pretreated groups after treatment with different concentrations of VP-16 (0–60 µM). *p<0.05 compared with the control group. All the experiments were repeated at least three times.

To study the expression of GRP78 protein in myofibroblasts, we used immunofluorescence technique to quantify the expression of GRP78. As shown in Fig. 3A, the green fluorescence of GRP78 displayed a perinuclear and reticular pattern of in the cells. Compared with the non-cancer induced cells, the intensity of the fluorescence in the myofibroblasts was obviously strong, which means lung cancer cells can stimulate the expression of GRP78 in fibroblasts significantly (Fig. 3B, p<0.05).

### Correlation between Over-expression of GRP78 and Drug Resistance to VP-16

In order to test whether the elevated expression of GRP78 in myofibroblasts cells is associated with resistance to VP-16, apoptotic assay was done using a range of VP-16 concentrations to evaluate the drug sensitivity of the two groups of cells. As shown in Fig. 2C and Fig. 2D, compared with non-induced cells, the myofibroblasts showed dramatic drug resistance to VP-16 at different concentrations with dose dependent manner (15, 30, 45, 60 µM) (p<0.05). These results suggested that GRP78 over-expression could suppress VP-16-induced apoptosis in myofibroblasts.

Furthermore, to test whether inhibition of the activities of GRP78 by EGCG could lead to the reviving of VP-16 sensitivity to VP-16 in myofibroblasts, the percentage for the apoptotic cells with or without the pretreatment of EGCG was measured. After exposure to VP-16 and staining with Hoechst 33342/PI, characteristics of apoptosis were demonstrated. As shown in Fig. 3C and Fig. 3D, the percentage apoptotic cells of EGCG-pretreated group was higher than that of non-EGCG pretreated group exposing to different concentrations of VP-16 (p<0.05), suggesting that the inhibition of GRP78 by EGCG could lead to the recovery of chemosensitivity to VP-16 in myofibroblasts.

## Discussion

A growing body of evidence suggests that activated fibroblasts, the main tumor stroma cells, are key determinant in malignant progression of cancer and represent an important target for cancer therapies [5], [6], [7]. So, in this study, we described a straightforward microfluidic device for the investigation of the response of myofibroblasts induced by lung cancer cells to anticancer drug VP-16 and the chemoresistant mechanism associated with GRP78 up-regulation.

Our microfluidic device consists of a double-layer chip and an injection pump. Using this device, lung cancer cells in 2D mode and fibroblasts in 3D mode were co-cultured to study the cellular response of fibroblasts with indirect contact. In vivo, cancer cells also grew in 3D as the fibroblasts and could actively influence their adjacent stroma by producing stroma-modulating growth factors. However, for our work here, the mainly purpose is to investigate the function of myofibroblasts after being induced by the soluble factors cancer cells secreted, so we just embedded the fibroblasts in 3D matrix mimicking the specific 3D feature. As for the cancer cells, since we only need their cytokine secretion, and it has been demonstrated that there was no difference in the amounts and quality of soluble factors between 2D and 3D culture modes for cancer cells [22], [23], and also, compared to 3D mode, it is more easily and freely for the soluble factors to flow for 2D mode, thus, we cultured them in 2D mode. Our results also demonstrated that this co-culture set-up and the co-culture mode could reflect the interaction between the two types of cells in vivo exactly. Compared to the conventional methods, our device has the following advantages. First, a pump was used to supply and transfer media, buffers, and even air, and waste products from cellular activities were drained, thus resembling the function of human circulatory system. Microfluidics technology can mimic a microenvironment faithfully and facilitate the study of cell behavior in vitro because it provides continuous medium to cells as in vivo micro-physiological microenvironment [24]. Second, as the scale of the fluidic micro-volume is roughly proportional to living cell sizes and can be upgraded with micro-processing capabilities, combinations of biological means and micro-electromechanical systems are useful to achieve practical prototypical micro-devices, such as tools for investigating cellular functions and establishing bio-microreactors [25], [26]. For example, an enclosed environment of the microfluidic systems is beneficial for preserving the activities of cytokines. Third, to investigate the function of fibroblasts in a physically relevant 3D microenvironment, we embedded fibroblasts in matrix, receiving the soluble factors secreted by cancer cells continuously. It has been supported in reports that cells embedded in 3D matrix represent real morphogenesis and gene expression profiles that closely resemble the in vivo biological activities [27], [28].

Our investigation revealed several interesting issues. First, the cytokines from lung cancer cells effectively transformed the co-cultured fibroblasts into myofibroblasts by indirect contact. Second, the expression of GRP78 in myofibroblasts could be elevated by the induction of lung cancer cells, and the up-regulation of GRP78 could protect the cells from apoptosis induced by VP-16. Third, the function of GRP78 could be inhibited by EGCG and this inhibition could revive the sensitivity to VP-16 in myofiroblasts. All these suggested that over-expression of GRP78 in myofibroblasts was associated with chemoresistance to VP-16. In order to investigate whether this up-regulation of GRP78 in myofibroblasts induced by lung cancer NCI-H460 cells is only limited to one type of the cells, we co-cultured another type of lung cancer cell line SPCA-1 (The Cell Bank of Type Culture Collection, Shanghai, China) and fibroblast cell line HFL1 on this device as well. The results showed that the α-SMA and GRP78 level were abundantly increased for HFL1 after being indirect contact co-cultured with the SPCA-1 cells, while only mild α-SMA and GRP78 expression was detected when cultured with itself alone (data was not shown). The mechanisms causing this effect are being studied. To our best knowledge, this is the first report of up-regulation of GRP78 in VP-16 chemoresistance in myofibroblasts induced by lung cancer cells.

Accumulated evidence indicates that the elevation of GRP78 is associated with the resistance to VP-16 [29], [30], [31], [32].GRP78 is a representative endoplasmic reticulum chaperone and could be up-regulated under stress conditions, such as glucose deprivation, hypoxia, or the presence of toxic agents [33], [34], all of which are found in the tumor microenvironment. It is now clear that VP-16 inhibits topoisomerase II through stabilizing the TopoII-DNA complex, preventing the rejoining of DNA strands, which inhibits DNA synthesis, and exerting cytotoxic effects [35], [36]. It was proposed that TopoII expression is lower in GRP78-overexpressing cells [37]; thus, it was possible that the myofibroblasts induced by lung cancer cells exhibited a decreased TopoII expression, resulting in the development of resistance to VP-16. Furthermore, GRP78 inhibits the activation of caspase-7 components, as well as suppression of the cleavage of procaspase-7 [30], [38], [39]. Therefore, activation of caspase-7 induced by VP-16 may be inhibited by GRP78 over-expression, leading to chemoresistance in myofibroblasts.

We previously reported that GRP78 level was related to the differentiation and progression of lung cancer and the VP-16 resistance was correlated to over-expression of GRP78 in human lung cancer cell lines [32], [40], [41]. In this study, we showed that up-regulation of GRP78 induced by cancer cytokines could confer the chemoresistance to VP-16 in myofibroblasts as well. This implies that drugs that target GRP78 expression and/or activity could potentially complement cancer therapy to eliminate residual tumors. Recently, it was discovered that EGCG, a major component of green tea, can bind to intracellular GRP78, inhibit its protective function, and increase chemosensitivity to VP-16 in breast and bladder carcinoma cell lines [21]. In our study, we showed that the myofibroblasts treated with the combination of EGCG and VP-16 exhibited more cell apoptosis than cells treated with VP-16 alone. All these results suggested that blocking the activity of GRP78 would significantly enhance both lung cancer cells and myofibroblasts susceptibility to anticancer drug VP-16, thereby eliminating both the lung cancer cells and its surrounding stroma.

In summary, we described a simple microfluidic device for the investigation of the response of myofibroblasts induced by lung cancer cells to anticancer drug VP-16. Our discovery of the relationship between GRP78 and myofibroblasts chemoresistance to VP-16 will open new directions in anticancer therapies for lung cancers. The methodology reported is straightforward and easy to operate. It can be used not only to investigate the cellular events between fibroblasts and cancer cells, but can also provide an effective method for interaction between multiple heterotypic cells and mimic the sophisticated microenvironment in vivo.
